# *sart-3* functions to regulate germline sex determination in *C. elegans* .

**DOI:** 10.17912/micropub.biology.000820

**Published:** 2023-05-02

**Authors:** Tokiko Furuta, Swathi Arur

**Affiliations:** 1 Department of Genetics, University of Texas MD Anderson Cancer Center

## Abstract

*Caenorhabditis elegans* gene *sart-3* was first identified as the homolog of human SART3 ( S quamous cell carcinoma A ntigen R ecognized by T -cells 3). In humans, expression of SART3 is associated with squamous cell carcinoma, thus most of the studies focus on its potential role as a target of cancer immunotherapy (Shichijo et al. 1998; Yang et al. 1999). Furthermore, SART3 is also known as Tip110 (Liu et al. 2002; Whitmill et al. 2016) in the context of HIV virus host activation pathway. Despite these disease related studies, the molecular function of this protein was not revealed until the yeast homolog was identified as spliceosome U4/U6 snRNP recycling factor (Bell et al. 2002). The function of SART3 in development, however, remains unknown. Here we report that the *C. elegans* *sart-3* mutant hermaphrodites exhibit a Mog ( M asculinization O f the G ermline) phenotype in adulthood suggesting that *sart-3* normally functions to regulate the switch from spermatogenic to oogenic gametic sex.

**Figure 1. Deletions in C. elegans sart-3 result in a Mog ( M asculinization O f the G erm line) phenotype:**
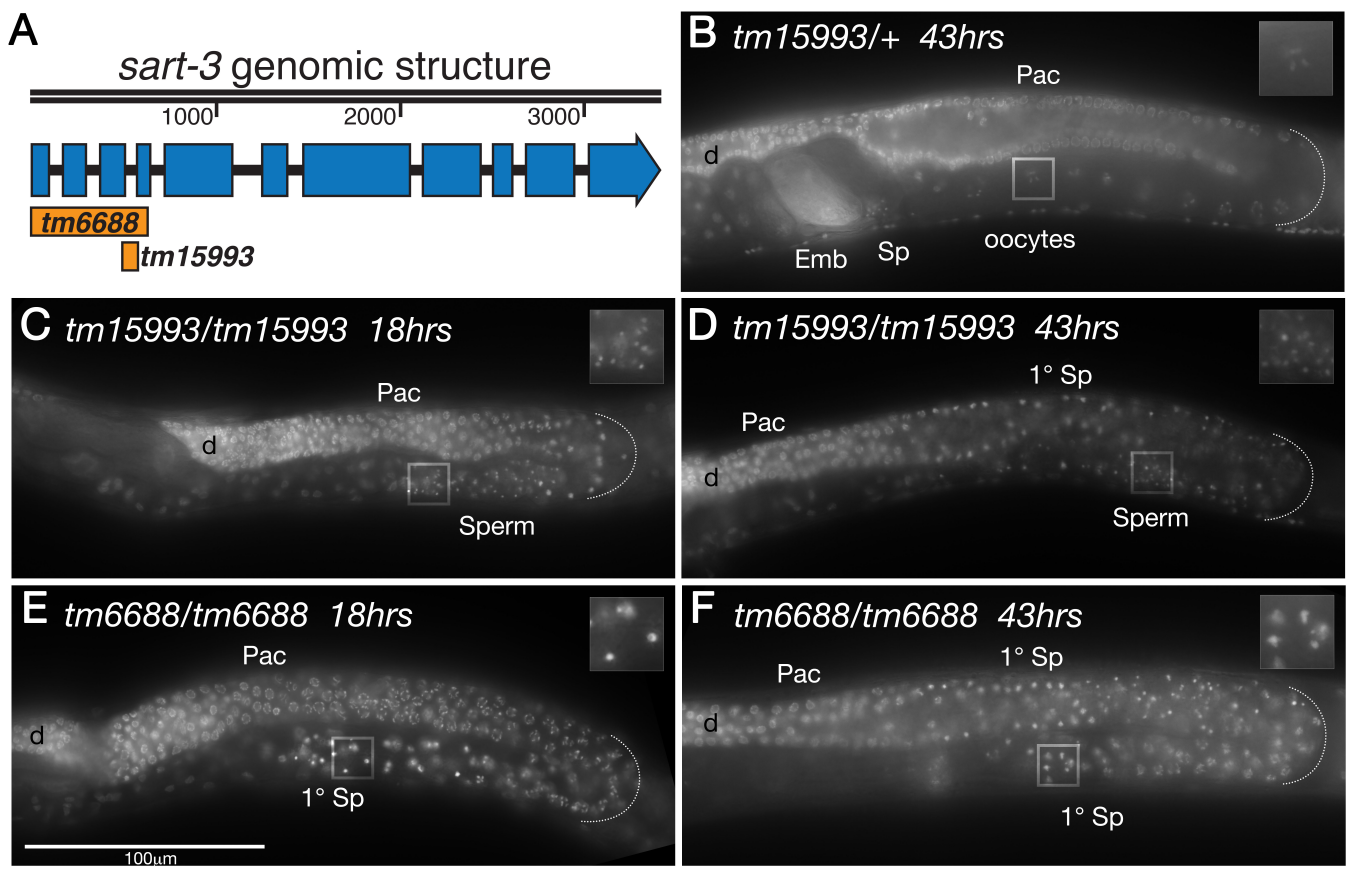
(A) Genomic structure of *sart-3* . Blue boxes indicate exons and black solid line indicates introns. *sart-3* gene spans 3.4kb in the genome. Two available deletion mutants are indicated with orange bars. Scale bar denotes base pairs. (B-F) Whole mount DAPI stained adult hermaphrodite germlines oriented with a distal to proximal polarity with respect to the spermatheca (Sp). (B) *tm15993/+* heterozygous adult control animal grown for 43hrs past L4 stage of development at 20°C exhibits oocyte development in the proximal germline. Inset shows DAPI stained diakinetic nuclei of the oocytes. (C-D) *tm15993* homozygous adult hermaphrodite germlines exhibit male germ cells that have completed meiotic divisions and form mature sperm (Sperm). Inset shows enlarged DNA morphology stained by DAPI. (E-F) Adult *tm6688* homozygous hermaphrodite germlines exhibit only the presence of primary spermatocytes (1°Sp). Mature sperm is not observed in this mutant. (C, E) 18hrs past L4 stage of development at 20°C. (D, F) 43hrs past L4 stage of development at 20°C. Emb: Embryos, Sp: Spermatheca and sperm, 1°Sp: Primary Spermatocyte, Sperm: Spermatids, d: distal most part of gonad shown in the micrograph, pac: pachytene stage germ cells, dotted line: indicates loop region. Inset: Enlarged indicated regions show the nuclear morphology as visualized by DAPI staining. The heterozygous germline shows bivalent nuclei in an oocyte, the homozygous mutant shows either condensed primary spermatocytes or spermatids that have completed two rounds of meiotic divisions.

## Description

To determine the function of *C. elegans* *sart-3* (renamed as S pliceosome A ssociated R na (RNA) binding fac T or), we obtained two deletion alleles from the National Bioresource Project (NBRP) for *C.elegans* , Japan and characterized the phenotype of the homozygous alleles. The *sart-3* *tm6688* allele deletes exons 1- 4, removing 645bp of the 5’ end of the gene including the start codon. As the deletion removes the 5’ end of the gene, we assume that *tm6688* is a genetic null allele. *tm15993* allele is a deletion which removes 82bp genomic region including 41bp of the coding region between exon 3 and exon 4. The tm15993 deletion creates a frameshift that results in the generation of three new amino acids before resulting in a stop codon, eventually leading to a truncated protein (Figure).

Homozygous mutants of each allele displayed 100% sterility (n>20). The heterozygous animal from each allele presents with normal brood size (~340). However, during the maintenance of the animals at 20 ^o^ C, we observed that the *tm15993* mutants displayed fewer homozygous adults relative to the *tm6688* mutants. To investigate whether the *tm15993* homozygous animals were lethal during development, we performed the following analysis. 27hrs past L4 heterozygous *tm15993* adult animals (P0) were allowed to deposit embryos for 18hrs at 20 ^o^ C and then removed from the plates. The embryos deposited on the plate were allowed to develop at 20 ^o^ C, and we assayed for (i) whether they hatched and (ii) the hatched progeny developed to adulthood. An inability to hatch was defined as embryonic lethality and an inability to reach to adulthood was defined as larval lethality. We observed that of the 694 F1 progeny analyzed from the *tm15993/tmC9* heterozygous mother, 522 were GFP positive. This GFP positive ratio corresponds to progeny numbers that are *tm15993* heterozygous as well as progeny that are homozygous for the balancer only, totaling to 75.2% of the population as would be expected from the Mendelian ratio. As the remaining progeny were negative for the balancer, they are homozygous mutants and correspond to 24.8% of the total progeny deposited on the plate, suggesting that the *tm15993* homozygous mutants are not embryonic lethal. All the F1 worms, now in a late larval stage on the test plate were counted and transferred to a new plate for easier scoring. These F1 progeny were allowed to develop for 24hrs at 20°C and scored for survival to adulthood. During this process, we noticed that 12 animals had likely crawled off the plate since we could only find 160 worms including dead larvae / adults to score at the end of experiment. We observed that 149 of the 160 animals were either sick or dead, suggesting that 93.1% F1 homozygous *tm15993* animals were larval lethal. 11 of the 160 animals survived to adulthood and presented with a sterile phenotype. These data suggest that only 1.7% of the total population that are homozygous for *tm15993* survive to adulthood. In contrast, a similar analysis with the *tm6688* allele revealed that 21.1% of the F1 progeny from a heterozygous mother were homozygous that survived to adulthood. 21.1% is not significantly different from the expected 25% by Mendelian ratio (P=0.3613, Chi-square test). Thus, we reasoned that the *tm15993* allele but not the *tm6688* allele displays larval lethality which leads to fewer homozygous adult animals in the *tm15993* mutant. The difference in the presence of larval lethality phenotype between the two alleles suggests that *tm15993* may either carry a linked mutation in the background despite being backcrossed five times, or it is likely that the truncated product is somehow toxic to larval development. In either case, because the common phenotype between the two alleles is sterility, we proceeded with characterizing this phenotype.

To determine the nature of sterility of the alleles, L4 hermaphroditic worms from *sart-3 (tm15993)/tmC9* or *sart-3 (tm6688)/tmC9* were incubated for 18hrs at 20°C and analyzed by wholemount DAPI staining (Methods). WT hermaphroditic animals at 43hrs past L4 exhibit formation of oocytes in the proximal arm (Figure, Panel B), with mature sperm stored in the spermatheca. Surprisingly, however, we observed that the homozygous *sart-3* animals from both the alleles did not develop oocytes and instead exhibited only the presence of sperm or spermatocytes, suggesting that the mutant animals did not switch to the oogenic sex in adulthood. This is a well characterized phenotype termed M asculinization O f the G erm line (Mog) (Hodgkin 1987) . To assess whether the germline failed to switch to the oogenic gametic sex or was just delayed in executing this switch, we let the animals develop for an additional 25hrs (so a total of 43hrs). At 43hrs past L4 stage of development, we observed that the mutant animals continued to display the Mog phenotype, suggesting that the germ cells were not just delayed in the formation of oocytes but in fact did not switch from a spermatogenic to oogenic gametic sex. This phenotype is consistent with the previous finding that reduction of many members of spliceosome machinery results in a failure to regulate the switch in gametic sex and leads to a Mog phenotype (Kerins et al. 2010) . As *sart-3* is identified to be an extended member of the U4/U6 spliceosome family, we propose that this work identifies a new member that functions with the conserved spliceosome machinery to regulate gametogenesis and thus phenocopies some of the known members of the spliceosome pathway.

## Methods

Whole mount DAPI staining: Harvested worms were washed three times with 1xPBS with 0.1% Tween20 (1xPBST) and fixed in 3% paraformaldehyde (made in 0.1M Potassium phosphate buffer, pH 7.2) for 15min at room temperature *.* After washing three times with 1xPBS, samples were post fixed with 100% Methanol containing 100ng/ml DAPI for 30 min or more at -20°C. Samples were then washed with 1xPBS a total of three times and mounted onto 4% agarose pad for imaging.

Imaging: Images were taken on the Zeiss Compound microscope Axio Imager.M2, using EC PLAN NEOFLUAR 40X/1.3NA lens and Hamamatsu Flash 4.0 V3 sCMOS Camera.

## Reagents

*sart-3* deletion alleles: FX16561: *sart-3(tm6688) IV/nT1(qIs51)* *(IV;V)* and FX25993: *atm-1(tm5027) I; xpc-1(tm3886) sart-3(tm15993)/+ IV* were obtained from NBRP (Japan) and backcrossed five times with wild type N2 and *tmC9* to balance the chromosome. AUM1830: *sart-3(tm6688)/tmC9[* F36H1.2 *(tmIs1221[Pmyo-2::GFP])] IV* , AUM1863: *sart-3(tm15993)/ tmC9[* F36H1.2 *(tmIs1221[Pmyo-2::GFP])] IV* . Both alleles were verified by PCR and sequencing. The original *tm1599* 3 strain FX25993 contained deletions in *atm-1(tm5027) I* and *xpc-1(tm3886) IV* , we confirmed the removal of the of *xpc-1(tm3886)* deletion by sequencing in the final strain that was analyzed. The final strains will be available from CGC ( *C. elegans* genome Center).

*Primers:*

The following primer sequences were used to map the deletion breakpoints for the two mutant alleles.

TF172 *sart-3* pre F: TTATTTCTATAAAGCTTGCATGG

TF173 *sart-3* ex5 R: CTCAGAAACAAGCCTCTCGTG

WT amplicon size: 1249 bp

*tm15993* amplicon size: 1166 bp

*tm6688* amplicon size: 604 bp

## References

[R1] Shichijo S, Nakao M, Imai Y, Takasu H, Kawamoto M, Niiya F, Yang D, Toh Y, Yamana H, Itoh K (1998). A gene encoding antigenic peptides of human squamous cell carcinoma recognized by cytotoxic T lymphocytes.. J Exp Med.

[R2] Yang D, Nakao M, Shichijo S, Sasatomi T, Takasu H, Matsumoto H, Mori K, Hayashi A, Yamana H, Shirouzu K, Itoh K (1999). Identification of a gene coding for a protein possessing shared tumor epitopes capable of inducing HLA-A24-restricted cytotoxic T lymphocytes in cancer patients.. Cancer Res.

[R3] Liu Y, Li J, Kim BO, Pace BS, He JJ (2002). HIV-1 Tat protein-mediated transactivation of the HIV-1 long terminal repeat promoter is potentiated by a novel nuclear Tat-interacting protein of 110 kDa, Tip110.. J Biol Chem.

[R4] Whitmill A, Timani KA, Liu Y, He JJ (2016). Tip110: Physical properties, primary structure, and biological functions.. Life Sci.

[R5] Bell M, Schreiner S, Damianov A, Reddy R, Bindereif A (2002). p110, a novel human U6 snRNP protein and U4/U6 snRNP recycling factor.. EMBO J.

[R6] Hodgkin J (1987). A genetic analysis of the sex-determining gene, tra-1, in the nematode Caenorhabditis elegans.. Genes Dev.

[R7] Kerins JA, Hanazawa M, Dorsett M, Schedl T (2010). PRP-17 and the pre-mRNA splicing pathway are preferentially required for the proliferation versus meiotic development decision and germline sex determination in Caenorhabditis elegans.. Dev Dyn.

